# Editorial: Cardiovascular diseases related to diabetes and obesity - volume IV

**DOI:** 10.3389/fendo.2024.1458742

**Published:** 2024-07-18

**Authors:** Ying Xin, Huanhuan Wang, Lu Cai

**Affiliations:** ^1^ Key Laboratory of Pathobiology, Ministry of Education, Jilin University, Changchun, China; ^2^ Jilin Provincial Key Laboratory of Radiation Oncology and Therapy, The First Hospital of Jilin University, Changchun, China; ^3^ Pediatric Research Institute, Departments of Pediatrics, Radiation Oncology, Pharmacology and Toxicology, the University of Louisville School of Medicine, Louisville, KY, United States

**Keywords:** diabetic complications, cardiovascular diseases, obesity, hypertension, risk factor, diabetic cardiomyopathy

Obesity is a growing global health concern, with increasing rates worldwide. Studies suggest that global and severe obesity rates will reach 48.9% and 24.2%, respectively by 2030 (1). Obesity elevates the risk of developing diabetes mellitus (DM), dyslipidemia, hypertension (HTN), cardiovascular disease (CVD) (2), non-alcoholic fatty liver disease (3), and even mortality (4). Although these complications are incurable, they can be mitigated by lifestyle changes, increased physical activity, and a balanced diet. Understanding the pathogenesis of obesity- and diabetes-related CVD and developing early screening methods are critical to designing effective interventions. This Research Topic has produced a fourth volume with ten papers, including nine clinical studies and one review. These studies have revealed correlations between obesity-related indices and the risk of HTN and DM, identified the risk factors for diabetic cardiovascular complications, and built predictive models, offering new insights for the early detection of obesity-related diseases.

Obesity and related CVD are associated with an increase in mortality and all-cause mortality. Although a high body mass index (BMI) has been confirmed as a risk factor for CVD, it does not accurately reflect regional body fat distribution, and has limited predictive value for CVD risk. Therefore, Ren et al. conducted a prospective cohort study with 7,439 participants aged ≥45 years from China to assess a series of obesity indices (Chinese visceral adiposity index [CVAI], visceral adiposity index [VAI], a body shape index [ABSI], conicity index, waist circumference, and BMI) for predicting CVD using Cox proportional hazards regression analysis. The authors revealed that CVAI had a significantly higher predictive value than other obesity indicators. HTN is another major risk factor for CVD that interacts with obesity and DM. In a cross-sectional study of 5,127 individuals, Zhang et al. also found that the Metabolic Score for Visceral Fat, a new index based on laboratory and anthropometric measurements, was significantly and positively associated with the risk of developing HTN in a Chinese Han population. An imbalance in purine metabolism can lead to increased production of uric acid, known as hyperuricemia (HUA), which is a key feature of the metabolic syndrome that synergistically exacerbates the risk of CVD. Therefore, identifying effective predictors of HTN-HUA is essential to reducing the risk of CVD. Li et al. investigated the correlations between the atherogenic index of plasma (AIP), lipid accumulation product (LAP), VAI, triglyceride-glucose index, triglyceride-glucose index (TyG), body roundness index (BRI), ABSI, and cardiometabolic index (CMI) with HTN-HUA. Multifactorial logistic regression analysis showed significant associations of AIP, LAP, VAI, TyG, BRI, ABSI, and CMI with concurrent HTN-HUA. BRI stood out for its effectiveness and non-invasiveness, making it the first choice for early detection. Salivary alpha-amylase (sAA) is an enzyme essential for starch digestion in the oral cavity. sAA activity (sAAa) has been studied for its inverse correlation with obesity and insulin resistance. However, the complex associations between sAAa and adiposity markers related to CVD remained unknown. Al Akl et al. analyzed serum samples and clinical data from an obese/non-diabetic population in Qatar and found significant linear correlations between sAAa and obesity-related biomarkers including BMI, waist circumference, hip circumference, and high-density lipoprotein. Furthermore, they found that sAAa was significantly and positively correlated with lipocalin and negatively correlated with C-reactive protein, tumor necrosis factor-alpha, interleukin-6 and ghrelin, a series of chronic low-grade inflammation markers in overweight/obese adults. These four studies established the association between several obesity indicators and CVD and provided insight into their predictive value for CVD morbidity.

DM characterized by elevated plasma glucose levels can lead to macrovascular and microvascular complications, including coronary artery disease, peripheral arterial disease (PAD), cerebrovascular disease, and retinal and neurological disorders. Therefore, identifying effective screening indicators is important for risk stratification and early intervention for prevention. Historically, fasting plasma glucose has been a mainstay of monitoring in patients with diabetes, but more and more studies have highlighted its inaccuracy in predicting cardiovascular complications. Glycemic variability has received widespread attention. A *post hoc* analysis of the Action to Control Cardiovascular Risk in Diabetes study by Sheng et al. revealed that a 1.37-fold increased risk of adverse cardiovascular events was associated with higher glycated hemoglobin (HbA1c) variability, but not with higher fasting plasma glucose variability. Additionally, a retrospective analysis by Deng et al. from 2010–2023 found that patients with DM with HbA1c >7% had a higher risk of CVD compared with those with an early mean HbA1c ≤7%. Early treatment with SGLT-2 inhibitors or GLP-1 receptor agonists was associated with a significantly lower risk of CVD. Both studies suggest that HbA1c stabilization is important for the prevention of cardiovascular complications in patients with DM. PAD as a macrovascular complication of DM is commonly manifested by atherosclerosis with a greater susceptibility to cardiovascular (CV) events. It is often asymptomatic and leads to severe lower limb complications. Identifying risk indicators for PAD in patients with DM is crucial to predicting the occurrence of cardiovascular complications. Li et al. evaluated the predictive value of a new inflammatory indicator, the red blood cell distribution width-to-albumin ratio (RAR), for the risk of PAD. A weighted logistic regression model was developed using PAD as the outcome variable. Their findings, that a higher RAR was associated with an increased risk of PAD in patients with DM, underscore the potential utility of RAR in the diagnosis and treatment of PAD. Insulin resistance in DM contributes to vascular endothelial damage, promoting the formation of vulnerable plaques in the carotid artery and increasing the risk of ischemic stroke. Therefore, Liu et al. developed a nomogram model to predict ischemic stroke risk using carotid ultrasound, which displayed high diagnostic efficacy. Furthermore, Gong et al. developed an artificial intelligence model based on fundus images to predict the carotid intima-media thickness in patients with DM, and demonstrated its superior performance compared to conventional methods. These studies provide effective indicators and models for predicting and monitoring the occurrence of DM-related vascular complications, thus facilitating individualized interventions.

In addition, long non-coding RNAs involved in the regulation of gene transcription, mRNA stability, and translation efficiency, are also involved in the development of diabetic complications. Geng et al. summarized the regulatory role of long non-coding RNAs in the pathogenesis of diabetic complications from four pathogenic pathways: oxidative stress, inflammation, fibrosis, and microvascular dysfunction. They identified MALAT1 as the most remarkable potential biomarker and therapeutic target, offering new insights into the prevention and treatment of diabetic complications.

In summary, this Research Topic has provided new indicators for the prediction of obesity-related cardiovascular disease and diabetes complications, facilitating the understanding of the pathogenesis and early detection of these chronic diseases. Further clinical studies are still required in the future to prove the effectiveness of the indicators and to develop a new platform for individualized treatment regimens.

## Author contributions

YX: Conceptualization, Validation, Writing – original draft, Writing – review & editing. HW: Formal analysis, Investigation, Validation, Writing – original draft. LC: Conceptualization, Supervision, Validation, Writing – review & editing.
